# Mitochondrial stress response in lung cells triggered by the inhaled nanoplastics

**DOI:** 10.1007/s00204-025-04194-x

**Published:** 2025-09-26

**Authors:** Beata Siemiątkowska, Joanna Szczepanowska

**Affiliations:** Laboratory of Cellular Metabolism, Center for Basic and Translational Research in the Field of Biology and Biomedical Sciences, Nencki Institute of Experimental Biology, Polish Academy of Sciences, Warsaw, Poland

**Keywords:** Environmental stress, Nanoplastic, Lung, Mitochondrial stress, Oxidative stress

## Abstract

The lungs are the primary site of exposure to environmental stressors, making them particularly vulnerable to the effects of inhaled nanoplastic particles. Owing to their nanoscale size, nanoplastics penetrate deeper into the respiratory tract than microplastics do and are capable of interacting directly with alveolar cells. This review focuses on the impact of inhaling nanoplastic particles on mitochondrial function in lung tissue, particularly the activation of mitochondrial stress response pathways. Mitochondria, as central regulators of cellular energy and stress responses, exhibit heightened sensitivity to environmental stress. Many studies have shown that nanoplastic exposure disrupts mitochondrial functions, reduces the membrane potential, and induces oxidative stress, possibly causing inflammation and apoptosis. This review underscores the need for advanced research to understand the systemic effects of nanoplastics and their compounded toxicity when combined with other environmental pollutants. Studying the adaptive processes of mitochondria exposed to the stress of inhaled nanoplastics is particularly important because mitochondria are essential for life-supporting functions and cell fate decisions. Given that mitochondria are key cellular targets, studying their behavior may prove useful in finding strategies to reduce the health risks posed by nanoplastic inhalation.

## Highlights

- Environmental nanoplastics—dangerous stressors for human health; lung diseases
- Mitochondrial stress response impacts cellular bioenergetic state
- Nanoplastics affect mitochondrial stress response via oxidative stress
- Chronic nanoplastics stress on mitochondria in lung remains largely unexplored

## Background

Plastic pollution is one of the main environmental pollution factors of the twenty-first century. It poses a serious global environmental threat with irreversible impacts on terrestrial, aquatic, transitional, and human-made ecosystems and, importantly, on human health, particularly through the air route. In 2022, global plastic production reached 400.3 million tons and is expected to grow exponentially (Rafey and Siddiqui 2023).

The term ‘plastics’ includes biobased, organic, or engineered polymers. Plastics are developed using unrefined petroleum, flammable gas, and coal. Biobased plastics are made of raw materials such as carbohydrates and vegetable oil (Alhazmi et al. 2021).

Plastics in the environment can be broken down into smaller fragments, partially degraded into different chemicals, and completely broken down to CO_2_ (Dees et al. 2021). However, many non-biodegradable plastics endure in the environment for a prolonged period of time. Their size and shape can be modulated (Saha and Saha 2024), enabling more interactions with organisms, tissues, and cells and increasing potential health risks. These plastics contain a large variety of chemical compounds, such as polyvinyl chloride (PVC), polyethylene (PE), polyamide (PA or nylon), polystyrene (PS), polypropylene (PP), polyethylene terephthalate (PET), and high-density polyethylene (HDPE) (Gouin et al. 2024; Zaman and Newman 2021). Products made of these materials can degrade into micro- and nanoplastic particles. Thus, environmental micro/nanoplastics are heterogeneous (Alqahtani et al. 2023) and usually contain potentially toxic chemical additives (Sharma et al. 2023).

Nanoplastics are created when plastics undergo multiple degradation and disintegration processes, both biotic and abiotic. Physicochemical changes, mechanical breakdown, and UV exposure are known factors that contribute to the formation of micro- and nanoplastics (Okoye et al. 2022). Nanoplastics are typically defined as particles less than 1000 nm (1 µm) in diameter, although some definitions use 100 nm as the threshold (Gigault et al. 2018; Lambert and Wagner 2016).

The field of micro/nanoplastic research on human health is still relatively new, but there is growing concern about the impact of nanoplastics on human health. Many studies have demonstrated the accumulation of nanoplastics in the blood, organs, and even the placenta, linking this accumulation to inflammation (Ali et al. 2024; Marfella et al. 2024). Notably, nanoplastic particles (unlike microplastics) have been shown to cross the blood‒brain barrier in animals (Kopatz et al. 2023), and recent studies have indicated their accumulation in the human brain (Campen et al. 2024).

Because the lungs are the primary sites of exposure to environmental stressors, the inhalation of nanoplastic pollutants, rather than microplastics, plays a critical role in modulating mitochondrial function. Mitochondria are widely recognized as the primary sites of cellular energy production, generating the majority of cellular ATP (Abrahams et al. 1994; Mitchell 1961). They are multifunctional and form a critical control hub essential for maintaining cell and tissue homeostasis. Mitochondria also play a key role in various metabolic pathways, such as phospholipid biosynthesis, calcium regulation, and signaling pathways (Noji et al. 1997; Rizzuto et al. 1998; Vance and Tasseva 2013). In addition, mitochondria are the main sites where reactive oxygen species (ROS) are produced during oxidative phosphorylation. In properly functioning cells, ROS act primarily as signaling molecules, but when mitochondrial function is disrupted, ROS can contribute to various pathologies (McBride et al. 2006; Pearson et al. 2022).

This review aims to compile all current information on the impact of nanoplastics on mitochondrial function in lung tissue, with particular emphasis on the activation of the mitochondria–nucleus–mitochondria signaling pathway, known as the retrograde signaling pathway.

### Nanoplastic uptake and distribution within the respiratory system

The entry of nanoplastics into cells, their subsequent fate, and the resulting cellular consequences are influenced by factors such as the type of nanoplastic and its shape, size, and charge (Liu et al. 2021).

Currently, inhalation and digestion are the primary routes for the uptake of micro- or nanoparticles, unlike other possible entry methods (Ageel et al. 2022; Chen et al. 2020; Tunahan Kaya et al. 2018). Inhalation of nanoplastics into the human body occurs faster and more aggressively than does digestion, which is a rather slow process that allows detoxification in the liver. When microparticles are inhaled, they are largely eliminated from the respiratory system by airway mucus (Wang et al. 2021). However, interactions with mucus in the respiratory tract can have opposite effects on nanosized particles. Mucin, a key component of mucus, forms a corona around nanoparticles, which reduces their cytotoxicity and enhances their uptake by cells (Ji et al. 2021). Nanoplastic particles that reach bronchi and alveoli might be eliminated by pulmonary macrophages (Yue et al. 2022). Inhaled plastic particles first contact the lung-lining fluid and are then phagocytosed. Studies using simulated phagolysosomal fluid have shown that the content of this fluid influences cytotoxicity, particularly oxidative stress caused by treatment with nanoplastic particles (Baysal et al. 2024). Particles smaller than 10 µm can enter bronchioles, and particles smaller than 2.5 µm and ultrafine particles can penetrate alveoli (Kelly and Fussell 2012). The deposition efficiency is influenced by the size and, importantly, the shape of the particles (Islam et al. 2023). Nanoplastics and their toxic substances can be directly absorbed by the alveolar epithelium, leading to localized inflammation. Furthermore, nanoplastics can reach the bloodstream and lymph nodes (Liu et al. 2019) and can be transported throughout the body via the circulatory system (Khan and Jia 2023). Yet, there is limited quantitative data on nanoplastics’ deposition and redistribution in the human lungs. While most of the available studies focus on microplastic (Jenner et al. 2022; Vasse and Melgert 2024), there are computational studies on nanoplastics’ depositions (Huang et al. 2024) and the experimental studies are mostly qualitative and not quantitative, regarding presence, uptake and toxicity (Liu et al. 2022; Xu et al. 2019).

### Consequences of the nanoplastics exposure (inhalation)

Specific responses to nanoplastics exposure in pulmonary tissue include the activation of mitochondrial stress responses, which are critical for maintaining cellular homeostasis. Continuous inhalation of nanoplastics can cause diseases, especially among people working in conditions endangering them to these kinds of harmful factors (Pilou et al. 2016). Plastics pose a threat to human health by accumulating in the lungs, primarily in the form of microparticles and fibers (Pauly et al. 1998). Extended exposure to microplastics and nanoplastics is more dangerous for people with underlying health conditions, although even otherwise healthy individuals can experience asthma (Atis 2005) and pneumoconiosis (Turcotte et al. 2013).

Micro- and nanoplastic exposure via the respiratory tract can cause or worsen the development of respiratory diseases. For example, microplastics (no data on nanoplastics) affect the physiology of asthmatic mouse lungs, causing macrophage aggregation, inflammatory cell infiltration, and mucus formation. In this model, cellular stress increases and programmed cell death is activated (Lu et al. 2021). Nanoplastics were shown to promote damage in oral-nasal tissues, by causing macrophages cuproptosis (type of apoptosis) and elevated TNF-α secretion which activated a cascade leading to destruction of alveolar structure and development of emphysema (Bu et al. 2025). Other studies carried out on mice and the cell model BEAS-2B (immortalized cells derived from normal human bronchial epithelial cells) have shown that exposure to micro- and nanoplastics can increase the risk of chronic obstructive pulmonary disease (COPD) by causing protease‒antiprotease imbalance, mitochondrial dysfunction, ferroptosis, and ER stress (Dong et al. 2020; Yang et al. 2024). Further damage to mitochondria as a result of COPD is possible, as abnormalities in the lung epithelial cells of subjects with COPD reveal specific mitochondrial changes. Studies have shown a loss of cristae, abnormally branched mitochondrial networks, and swollen and fragmented organelles, which has been confirmed in long-term investigations (Hara et al. 2013; Hoffmann et al. 2013).

Idiopathic pulmonary fibrosis (IPF) is another respiratory disease closely linked to mitochondrial dysfunction. IPF is associated with reduced mitophagy, and its mitochondrial phenotype includes malformed mitochondria accumulation, decreased mitochondrial membrane potential (Δ*Ψ*), reduced electron transport chain (ETC) activity, increased ROS production, and increased mitochondrial permeability (Zank et al. 2018). Air pollution has been identified as a factor that exacerbates IPF’s clinical manifestations (Mariscal-Aguilar et al. 2024).

It was shown that in mice, inhalation of micro- and nanoplastics can cause changes in lung genes, promoting the expression of profibrotic genes (including Igkv14-126000 (a part of the immunoglobulin kappa variable), Egr1 (Early Growth Response 1), Scel (Sciellin), Lamb3 (Laminin Subunit Beta 3), and Upk3b (Uroplakin 3B)) (Jin et al. 2023). Epithelial‒mesenchymal transition, oxidative stress, changes in the mitochondrial membrane potential, and impaired cellular energy metabolism, as identified in A549 cells (human alveolar epithelial cells derived from lung adenocarcinoma), treated with nanoplastics also play important roles in the induction of pulmonary fibrosis (Halimu et al. 2022). It was observed that chronic inhalation of microplastics (no data on nanoplastics) caused fibrosis and even cancer in the lungs of rats (Borm 2022).

The human health risks associated with nanoplastics exposure remain insufficiently studied. However, the interaction between nanoplastics and mitochondria in respiratory system cells appears to represent a critical axis of toxicity. Therefore, by examining plastic-induced mitochondrial stress through mechanisms such as oxidative stress, mitochondrial membrane damage, and disrupted mitochondrial dynamics, we can demonstrate the cascading effects on the cellular and respiratory level.

## Stress response to nanoplastics exposure investigated in pulmonary models

### Cellular response depending on the size, concentration, charge, and shape of nanoplastics

Nanoplastics exposure impacts mitochondrial functions and dynamics in pulmonary cell models. The internalization of nanoplastics by cells is dependent on the size of particles. Smaller polystyrene nanoparticles, such as PS nanoplastics, with a diameter of 25 nm were internalized more rapidly than larger ones (70 nm) by A549 cells. Smaller particles have greater effects on the cell. Similarly, relatively small nanoplastics (25 nm) at relatively high concentrations (160 µg/ml) led to a relatively significant decrease in cell viability, with PS nanoplastics causing cell cycle S phase arrest, activating inflammatory gene transcription, and altering the expression of proteins related to the cell cycle and promoting apoptosis. Among the genes whose expression was altered by nanoplastic treatment were cell cycle-related genes such as those in the Cyclin D and E gene families (CCND1 and CCNE1), the proliferation marker Ki67, inflammation-related Interleukin 1 receptor type I (IL1R1), IL6 (Interleukin 6), and Interleukin 8 (CXCL8), which were significantly upregulated. Furthermore, transcription factors involved in inflammation, apoptosis, and survival, such as NF-κB, are activated. At the protein level, PS nanoplastics triggered apoptosis via the TNF-α-associated pathway. This included the upregulation of proapoptotic proteins such as Death Receptor 5 (DR5), Caspase-3, Caspase-8, Caspase-9, and mitochondrial apoptosis-related proteins like Cytochrome c. In addition, changes in the expression of cell cycle regulators such as Cyclin D3 and Cyclin E were observed (Xu et al. 2019). Numerous studies have shown that smaller plastic particles—nanosized—are more easily internalized, as shown in examples of many aquatic and terrestrial species and cells (Kögel et al. 2020; Liu et al. 2021).

In addition to size, the concentration of nanoplastics is another key factor influencing their internalization by cells. In A549 cells, as the concentration of nanoplastics increased from 2 to 125 μg/ml (at 136 nm), the concentration of ROS also increased. Moreover, higher concentrations of PET particles are more dangerous for cells and cause an increased level of DNA strand breaks, leading to genetic toxicity (Alzaben et al. 2023). The cellular localization of nanoplastics is also essential for the observed effects. A localization study of PS nanoplastics 50 nm and 500 nm in diameter was performed in A549 and BEAS-2B human lung cells. After 24 h of exposure, nanoplastics localized close to the mitochondria and in the lysosomes. After a longer incubation time, the nanoplastics are distributed from lysosomes to other regions of the body of the cell (Liu et al. 2022). HNEpCs (human nasal epithelial cells) exposed to 100 µg/ml for 24 h (ca. 100-nm particles) were localized in endosomes, surrounding nuclei, and inside nuclei (Annangi et al. 2023).

The effects of internalized plastic on cell viability have been described in several models. For example, in HNEpCs treated for 24 h with nanoplastics at concentrations ranging from 1 to 100 µg/ml, cell viability was not affected, which is in agreement with previous studies in Caco-2 cells (colorectal adenocarcinoma cell lines), HepG2 cells (liver cancer lines), and HepaRG cells (immortalized hepatic cells) (Stock et al. 2021). A slight but significant decrease in cell viability was observed in A549 cells, but only at very high concentrations of nanoplastics (98.4 and 196.72 µg/ml) (Zhang et al. 2022).

Another factor worth considering is the exposure time to nanoplastics. In the A549 cell line, after 48–96 h of exposure, across a range of doses (from 10 µg/ml to 500 µg/ml), a significant reduction in cell viability occurred. An increase in the ROS (H_₂_O_₂_) concentration was also observed (at all treatment doses), and 2 h after treatment, increased expression of genes associated with oxidative stress, including the mitochondrial isoform of superoxide dismutase (SOD2), was observed. Moreover, the long-term effects of stress caused by nanoplastics result in the induction of apoptosis and aging processes (Milillo et al. 2024).

A very important element that determines the interaction of a nanoplastics with a cell is its surface charge. An amine-modified polystyrene is positively charged and exhibits stronger electrostatic interactions with negatively charged cell membranes, leading to higher internalization rates, as shown in A549 and BEAS-2B (Clérigo et al. 2022; Halimu et al. 2022). Negatively charged nanoplastics, such as carboxylated polystyrene, show reduced uptake by A549 cells (Halimu et al. 2022). The coronas formed on positively and negatively charged nanoparticles differ. Proteins such as vitronectin and fibrinogen mediate their endocytosis. Vitronectin accumulates more on negatively charged particles, regardless of size, whereas fibrinogen prefers smaller (100 nm vs 300 nm), negatively charged particles. Negatively charged PS nanoplastics accumulate in the lungs of C57BL/6J mice (commonly employed in biomedical research because of their well-characterized genome, immune system, and behavioral traits) and are injected intravenously. Overall, the lung accumulation of negatively charged COOH-nanoplastics was significantly greater than that of NH2 nanoplastics, highlighting the impact of surface charge on in vivo behavior and biodistribution (Baimanov et al. 2024).

Negatively charged carboxylated plastic nanoparticles 20 nm in diameter cause the activation of basolateral potassium channels in human airway epithelial cells and persistent and concentration-dependent increases in short-circuit currents via the activation of ion channels and the stimulation of Cl^−^ and HCO^3−^ ion efflux (McCarthy et al. 2011).

The size, type, and concentration are often considered and prioritized in nanoplastics studies, but the charge often remains unaddressed. However, owing to aging or coating with organic and inorganic matter, neutral and weakly charged particles may dominate the airborne nanoplastics fraction (Aeschlimann et al. 2022). When considering the toxicity of the inhaled nanoplastics, we should take into account not only the experimental models used but also the properties and types of the nanoplastics. Moreover, use of more advanced study tools such as lung organoids could yield more relevant information. Up to date, such 3D culture mimicking organs are used very rarely, which is a gap in using advanced study tools in this field. Although, a recent study of nanoplastics in lung organoids has shown a disruption in normal cell differentiation and accumulation of transitional cells signaling impaired alveolar repair (Yang et al. 2025).

### Nanoplastics’ influence on mitochondrial function by activating the mitochondrial retrograde signaling

**Fig. 1:**
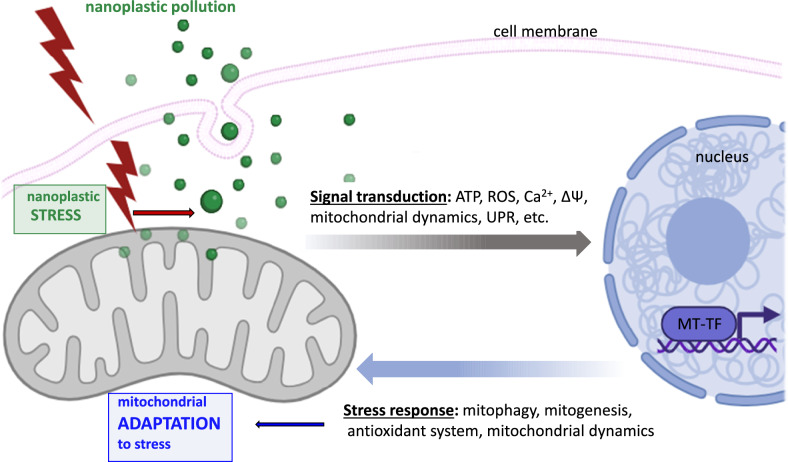
A general scheme of mitochondrial retrograde signaling pathway (mitochondria–nucleus–mitochondria). Mitochondria to nucleus signal transduction takes place by elements like, for example, ATP, ROS, calcium flux, mitochondrial membrane potential (Δ*ψ*), URPmt, mitochondrial network architecture, the signal is interpreted by nucleus and results in, e.g., transcription activation of specific “mitochondrial” genes, the stress response leads to changes in mitogenesis, mitophagy, mitochondrial network organization and finally to mitochondrial adaptation. Created with BioRender.com

A cell’s ability to consistently and reliably obtain, store, and utilize energy is fundamental to its survival, function, and overall health. Environmental stressors (such as nanoplastics) can target and interfere with mitochondrial function (Shaughnessy et al. 2014). The adaptation of mitochondrial functions to stress conditions requires a quick and flexible response, for which mitochondria–nucleus–mitochondria signaling, called retrograde signaling, is activated (Fig. 1).

Retrograde signaling appears to affect a wide range of processes, including the activation of many cellular signaling pathways (that regulate metabolic adaptation), antioxidant systems, cellular proliferation, apoptosis resistance, and cell death (Hijazi et al. 2020; Piantadosi and Suliman 2017). Mitochondrial stress results in the release of important second messenger stress signals (e.g., mitochondrial membrane potential (Δ*Ψ*), reactive oxygen species (ROS), Ca^2+^, NO, ATP/ADP, and mitochondrial unfolded protein (UPRmt)) that are transmitted to the nucleus (Walker and Moraes 2022). Subsequently, a signal from the nucleus to the mitochondria increases the mitochondrial stress response to maintain mitochondrial integrity by activating/remodeling the mitochondrial quality-control mechanisms responsible for the degradation of damaged/dysfunctional mitochondria (mitophagy), including the mitochondrial unfolded protein response, the dynamic mitochondrial network process, such as fission/fusion, mitochondrial biogenesis (mitogenesis), and the intracellular transfer of mitochondria. In other words, the nucleus‒mitochondrion response aims to adapt mitochondrial function to stressful conditions (Quirós et al. 2016).

Some elements indicating the activation of the mitochondria retrograde signaling pathway were observed after nanoplastics treatment in lung cells. Low levels of ROS are normally produced by mitochondria via incomplete oxygen reduction. An excess of ROS contributes to cellular oxidative stress, which in turn leads to disruption of mitochondrial dynamics and can ultimately contribute to apoptotic or necrotic cell death. As shown in studies using BEAS-2B and HPAEpiC cells (human pulmonary artery epithelial cells), elevated ROS levels are pivotal for polystyrene nanoplastics toxicity. A significant increase in superoxide anion (O2•─) and a decrease in the activity of antioxidant enzymes such as glutathione peroxidase (GSH-Px), catalase (CAT), and superoxide dismutase (SOD) occurred after treatment with 40-nm plastic beads at concentrations ranging from 7.5 to 30 µg/cm^2^ for up to 24 h. In addition to the activities of oxidoreductases, the levels of proteins such as Heme Oxygenase 1 (HO-1) and NAD(P)H Dehydrogenase [Quinone] (1NQO1) decreased. In addition, oxidized lipids accumulate in these lung cells, as indicated by increased levels of lipid peroxidation products such as MDA (Yang et al. 2021). Shi et al. investigated a mixture of polystyrene nanoplastics and phthalate esters in A549 cells. An increased level of total ROS and decreased SOD and CAT enzyme activity were detected at high concentrations of nanoplastics (200 µg/ml, 100 nm diameter) and microplastic (Shi et al. 2021). In the case of the A549 cell line, an increase in the ROS level was observed (at a nanoplastics concentration of 49.2 µg/ml) (Zhang et al. 2022).

Usually, an excess of ROS results in a decrease in the mitochondrial membrane potential (ΔΨ), which, as mentioned before, is one of the secondary messengers from the mitochondria to the nucleus. (Aguilar-Guzmán et al. 2022).

A study carried out in BEAS-2B cells using nanoplastics (100 nm, 60 µg/ml) revealed increased intracellular ROS with decreased Δ*Ψ* and increased Ca^2^⁺ levels (Xuan et al. 2023). Similarly, a significant decrease in the Δ*Ψ* was observed in HNEpCs treated with nanoplastics for 24 h (Annangi et al. 2023). In 2022, Lin reported that mitochondrial damage occurred in BEAS-2B cells exposed to 80-nm nanoplastics (at various concentrations). In this case, there was also increased mROS (mitochondrial ROS), decreased Δ*Ψ*, and inhibition of mitochondrial respiration. Interestingly, compared with liver cells, BEAS-2B cells are less vulnerable to nanoplastics treatment, especially at low concentrations (Lin et al. 2022).

In response to nanoplastics, after activation of signal transduction from the mitochondria to the nucleus, a nucleus–mitochondrion stress response pathway was also observed. The mitochondrial stress response manifests as changes in the dynamics of the mitochondrial network or in the process of biogenesis or mitophagy. Sometimes, excessive stress leads to mitochondrial disruption and cell death.

In HPAEpiC and BEAS-2B lung epithelial cells, Yang reported that polystyrene nanoplastics exposure activated the mitochondrial stress response pathway, leading to ferroptosis, a form of regulated cell death driven by oxidative stress. Mitochondrial ROS are pivotal in triggering iron accumulation and lipid peroxidation, leading to ferroptosis (Rochette et al. 2022). Ferroptosis was also shown to be induced by cationic nanoplastics in mice lung tissue by perturbing the core circadian transcription factors Bmal1. Bmal1 downregulation compromises antioxidant defenses, potentially involving NRF2 and HO-1 (Wu et al. 2025).

A study using both cell models and a mouse inhalation model demonstrated that 40-nm PS-NP treatment (at 7.5, 15, and 30 μg/cm^2^, with 7.5 μg/cm^2^ equivalent to 24 μg/ml) resulted in mitochondrial dysfunction. This was characterized by decreased energy production, increased proton leakage, and structural damage, including increased mitochondrial membrane density, fragmentation, and the disappearance of cristae. In addition, the treatment triggered cell autophagy (Yang et al. 2023). RNA-seq analysis revealed that differentially expressed transcripts in BEAS-2B cells exposed to PS nanoplastics were enriched in lipid metabolism and iron ion binding processes, with significant changes in the expression levels of transcripts encoding iron-related proteins. Wu et al. (2024) further confirmed that the HIF-1α/HO-1 (Hypoxia-Inducible Factor 1-Alpha/Heme Oxygenase 1) signaling pathway plays a crucial role in regulating ferroptosis in lung injury caused by PS micro- and nanoplastics exposure (Wu et al. 2024). These findings collectively demonstrate that the activation of the mitochondrial stress response pathway by PS nanoplastics exposure leads to cell death through ferroptosis in lung epithelial cells.

Nanoplastics affect mitochondrial network architecture. The shape of the mitochondria is tightly linked to their function, similar to the interactions they endure with other intracellular structures. The transition of mitochondrial morphology from elongated to fragmented alters and regulates mitochondrial function and plays an active role in the stress response pathway of retrograde signaling. Mitochondrial network dynamics are controlled by multiple proteins that mediate the remodeling of the outer and inner mitochondrial membranes and are engaged in the fusion/fission and transport of mitochondria (Picard et al. 2013). Although there are no data on nanoplastics affecting the mitochondrial network architecture in lung cells, data from other cell types are available. In HepG2 human hepatocellular carcinoma cells, treatment with nanoplastics (PS nanoplastics, diameter 21.5 ± 2.7 nm) at concentrations of 6.25, 12.5, 25, and 50 μg/ml for 24 h increased mitochondrial fusion and fission by increasing the level of the protein responsible for mitochondrial fission (DRP1) and decreasing the level of the protein involved in the fusion of the outer membrane of mitochondria with Opa1 (Li et al. 2023).

Similarly, in our study, Walczak et al. demonstrated that DRP1 is strongly affected by cigarette smoke (Walczak et al. 2020). Although several aspects of mitochondrial dynamics have been explored or implicated in the context of nanoplastics exposure, the primary targets of disturbance, particularly the processes of fusion and fission, remain largely unknown.

Changes in mitochondrial architecture are also present in the process of biogenesis and mitophagy.

Mitochondrial biogenesis, a crucial component of retrograde signaling (on the way nucleus – mitochondria), involves the coordinated expression of nuclear and mitochondrial genes (mitochondria have their own DNA) (Couvillion et al. 2016; Diaz and Moraes 2008; Lee et al. 2018). The transcriptional coactivator peroxisome proliferator-activated receptor gamma coactivator 1-alpha (PGC-1α) activates the main mitochondrial transcription factor nuclear respiratory factor 1 and 2 (NRF1/2), which regulates the expression of genes essential for mitochondrial function (including those involved in oxidative phosphorylation (OXPHOS) and mitochondrial DNA replication) (Biswas and Chan 2010; Gleyzer et al. 2005; Gleyzer and Scarpulla 2011; Sun et al. 2023). Unfortunately, the process of mitochondrial biogenesis has not been studied in the lung after nanoplastic treatment. To date, studies have been performed using a mouse model and AML-12 hepatocytes. Different dosages of 20 nm nanoplastics caused excessive production of ROS and repressed nuclear NRF2 (Wen et al. 2024). We have previously demonstrated by experiments performed in BEAS-2B cells that changes occur in mitochondrial morphology and network, and that biogenesis markers decrease, upon treatment with the total particulate matter from cigarette smoke and aerosol from the tobacco heating system (Walczak et al. 2020). Similar processes may occur in mitochondria following nanoplastics exposure. This knowledge gap warrants further investigation.

To maintain mitochondrial homeostasis within the cell, the balance of mitogenesis and mitophagy is essential. Mitophagy clears damaged mitochondria. (Lampert et al. 2019; Sekine and Youle 2018; Vives-Bauza et al. 2010). To date, no studies have directly investigated the effects of nanoplastics on mitochondrial mitophagy in lung tissue. However, in HNEpCs, co-localization of the LC3-II protein and nanoplastic particles, even LC3-II protein expression, was not observed via Western blotting and via confocal microscopy (Annangi et al. 2023). LC3-II (microtubule-associated protein light chain 3-II) is considered the primary autophagy marker; yet, most mitophagy-related proteins contain LC3-interacting region (LIR) motifs, which can directly interact with LC3 to promote the engulfment of defective mitochondria (J. Sun et al. 2022). Alterations in mitochondrial fusion, fission, and mitophagy are linked to the pathophysiology of many diseases, among them neurodegenerative diseases such as Parkinson’s disease, Alzheimer’s disease (AD) and amyotrophic lateral sclerosis (ALS), as we have previously pointed out (Drabik et al. 2021a, b; Kamienieva et al. 2021) and as we implicated as nicotine-caused possible damages (Malińska et al. 2019). Hence, investigating how nanoplastics affect these mitochondrial processes is likely to yield important insights into their detrimental impact on mitochondrial dynamics and quality control, addressing the present gap in our understanding of nanoplastics–mitochondria interactions.

Some studies have provided indirect evidence that nanoplastics have an impact on mitochondrial function. Nonetheless, we suggest these effects are consequences of the mitochondrial stress response, leading to adaptation of both cell mitochondrial as well as cell.

Experiments on A549 and THP-1 (human monocytic cells) cells with heat-aged nanoplastics (nanoplastics that have undergone a thermal aging process, which alters their physicochemical properties and potentially affects their toxicity) revealed the effects of TNF-α (tumor necrosis factor-alpha) on mitochondria. Increased ROS production, the activation of apoptosis, and alterations in mitochondrial dynamics have been shown. In THP-1 cells, nanoplastics increase the level of IL-1β, and inflammasome activation and ultimately mitochondrial damage are observed (Antonio et al. 2024). Another study on A549 cells revealed that larger nanoplastics (202 nm and 535 nm) induced a stronger inflammatory response than did smaller nanoplastics (64 nm), as indicated by increased IL-8 expression (Brown et al. 2001). Despite the increase in ROS, PS nanoplastics do not consistently induce inflammation. In A549 cells treated with 100-nm nanoplastics (Laganà et al. 2024) and BEAS-2B cells treated with 50-nm nanoparticles (Gosselink et al. 2024), the treatment failed to elicit a significant inflammatory response, as measured by the secretion of markers such as IL-8, in these cell lines.

In short, in lung cells, nanoplastics exposure induces mitochondrial stress and ROS, activating retrograde signaling to the nucleus, including the mitochondrial unfolded protein response (UPRmt), which regulates antioxidant and quality-control genes. The nucleus reciprocally modulates mitochondrial function via anterograde signals controlling biogenesis and protein expression, with cell-type differences. However, significant knowledge gaps remain. Direct evidence of retrograde signaling activation in lung cells upon nanoplastics treatment is still lacking, particularly regarding UPRmt-specific markers and transcriptional regulators such as ATF4/5, NRF2, or CHOP. The impact of nanoplastics on mitochondrial biogenesis (PGC-1α/NRF1/2/TFAM axis) and on key mitophagy pathways (PINK1, PARKIN, BNIP3) has not been directly addressed in lung cells. Similarly, the regulation of mitochondrial fusion and fission proteins (DRP1, MFN1/2, OPA1) under nanoplastics stress is unknown. Addressing these gaps will be crucial for establishing a comprehensive view of how nanoplastics impair mitochondrial–nuclear communication in lung cells.

### Nanoplastic effects: cellular response specific to pulmonary tissue

Some studies have shown that lung tissue responds to nanoplastics in a characteristic way. Pulmonary cells often exhibit increased levels of enzymes such as SOD and CAT, which mitigate the oxidative stress caused by nanoplastics (Lai et al. 2022; Mahmud et al. 2024). Respiratory epithelial cells are key orchestrators of pulmonary innate immunity, suggesting that they have enhanced defensive capabilities, including antioxidant mechanisms (Whitsett and Alenghat 2015). This is in accordance with a study by Alzaben et al. (2023). A study on model lung epithelial cells using polyethylene terephthalate (PET) nanoparticles revealed an increase in intracellular reactive oxygen species (ROS) production with increasing nanoplastics concentrations. However, the increase was relatively low compared with that of other cell-type controls (Alzaben et al. 2023). Furthermore, the varying susceptibility of the models shapes our understanding of the effects of plastic on the lungs and other cell types. Owing to their ability to promote continuous proliferation, cancer-based models may not accurately reflect nanoplastic toxicity. Stem cell-derived, tissue-like cells, which maintain greater physiological proliferation, offer a better representation of these toxic effects (Busch et al. 2023). Therefore, understanding the impact of nanoplastics on mitochondrial functions is important because it could be crucial for accurately assessing their toxicity to cells, tissues, and organisms.

## Summary and conclusion

The studies mentioned above highlight the significant impact of nanoplastics on the functioning of mitochondria in lung cells, which are directly exposed to this external stressor.

Mitochondria are central to the cell responsible for energy production and the stress response. Disfunction of mitochondria has been linked to many diseases, such as age-related diseases, neurodegenerative, cardiac and metabolic diseases, cancer, and senescence, chronic mitochondrial damage, which can lead to death. Mitochondria can respond to stressors to support cell survival or death through energy production and the activation of signaling pathways. Therefore, studying their behavior may prove useful in finding strategies to reduce the health risks posed by nanoplastics inhalation.

**Fig. 2:**
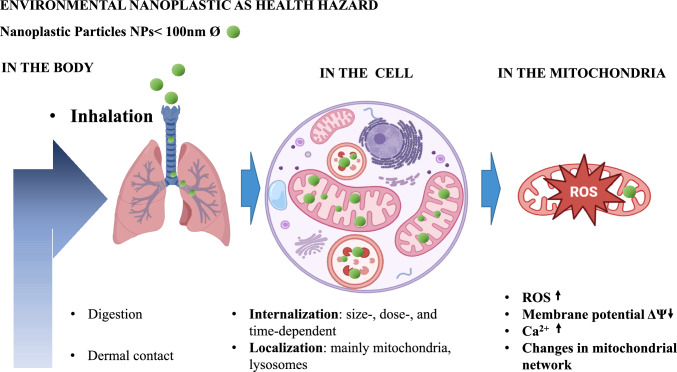
The nanoplastic pathway: from environment to mitochondria. The nanoplastic pathway: from environment to mitochondria. Nanoplastic range of size is particle diameter below 100 nm, entry points for it are by inhalation, ingestion, and dermal contact, from most to the least prominent gateway. These particles enter cells in a manner dependent on their size, dose, and shape, smaller particles enter more efficiently than bigger. They localize in or near mitochondria and lysosomes, from where they can be redistributed through the cell. Effects of the nanoplastics: in the cell—reduction of viability and proliferation, calcium overload; in the mitochondria—oxidative stress, reduction of membrane potential, and activation of mitochondrial retrograde signaling leading to adaptation. Created with bioRende.com

In conclusion, knowledge about the effects of nanoplastics (short- and long-term) on cellular energy metabolism and mitochondrial behavior in the lungs is insufficient (Fig. 2).

For the time being, nanoplastics are known to cause oxidative stress in lung tissue, contributing to inflammation and inducing apoptosis.

This is still not enough to demonstrate the entire path of the mitochondrial response and bioenergetic adaptation to chronic or short-term stress. Moreover, the response of mitochondria to stress or their malfunctions not only exacerbates the onset of pulmonary diseases but also highlights the potential systemic risks of inhaled nanoplastics, which may spread through the bloodstream to other organs. Future research is essential to elucidate the effects of nanoplastics on mitochondrial function and develop targeted interventions to mitigate the effects of inhaled nanoplastic particles on human health. This is particularly important as, despite growing regulatory efforts at global levels to reduce plastic pollution, atmospheric nanoplastic levels continue to rise, highlighting the need for comprehensive scientific understanding and effective mitigation strategies.

## Future perspectives

The lack of quantitative and systematic data on nanoplastics in the air and lack of qualitative data on nanoplastics deposition and clearance in the respiratory system is a deficit in knowledge influencing the design of current and future experimental in vitro research. Moreover, this deficiency in comprehension prohibits specifying the thresholds for toxicity in different study models and in human-relevant exposure scenarios. To further develop the nanoplastics inhalation research area, studies should include comparative analysis in different lung cell types and more advanced models (such as lung organoids or animals).

In the context of metabolism and behavior of mitochondria in lung cells, several important questions remain to be addressed, namely about the dosages and treatment times leading to adaptation *vs*. irreversible damage. A complete mapping of retrograde signaling pathway in lung cells after nanoplastics exposure: (1) signal transduction parameters from mitochondria to nucleus, (2) response pathway in retrograde signaling; mitochondrial biogenesis, mitophagy and mitochondrial dynamics network morphology, is essential to understand the most important way of cellular adaptation.

Investigating mitochondrial function under stress induced by nanoplastics pollution may provide critical insights into the mechanisms by which cells adapt their bioenergetics properties to altered environmental conditions. Given the ubiquitous presence of nanoplastic particles in our environment, elucidating the metabolic reprogramming of cells is essential for identifying pathways that support adaptation as well as those that trigger severe pathologies or result in cell death.

For many years, our research has focused on mitochondrial stress in various pathological states of the cell, including cancers (Walczak et al. 2017; Wojewoda et al. 2010, 2011, 2012) and neurodegenerative diseases such as amyotrophic lateral sclerosis, Alzheimer’s disease, and Parkinson’s disease (Drabik et al. 2021a, b; Kamienieva et al. 2021, 2023; Walczak et al. 2019). We have also investigated mitochondrial stress induced by cigarette smoke pollutants in lung cells (Walczak et al. 2020; Malinska et al. 2018; Malińska et al. 2019). Currently, our studies are focused on elucidating the effects of nanoplastics on both normal lung cells and lung cancer cells on activation and functioning of mitochondrial retrograde signaling leading to adaptation.

In summary, given the growing importance of research on the impact of nanoplastics on human health, we are deeply committed to advancing the understanding of their effects on retrograde signaling and cellular adaptation in such conditions.

## Data Availability

The data supporting the findings of this study are available from the corresponding author upon reasonable request.

## Abbreviations

1NQO1: NAD(P)H Dehydrogenase [Quinone]
A549: Adenocarcinomic human alveolar basal epithelial cells
ADP: Adenosine diphosphate
AML-12: Alpha mouse liver 12
ATP: Adenosine triphosphate
BEAS-2B: Bronchial epithelium transformed with Ad12-SV40 hybrid virus
C57BL/6 J: C57 Black 6 Jackson mice
Ca^2^⁺: Calcium ion
Caco-2: Colorectal adenocarcinoma cell line
CAS: Caspase
CAT: Catalase
CCND1: Cyclin D
CCNE1: Cyclin E1
Cl-: Chloride ion
CO2: Carbon dioxide
COOH-NPs: Carboxylated nanoplastic particles (negatively charged)
COOH-PS: Carboxyl-functionalized polystyrene nanoparticles (negatively charged)
COPD: Chronic obstructive pulmonary disease
CXCL8: Interleukin 8
Cyt c: Cytochrome C
DNA: Deoxyribonucleic acid
DR5: Death Receptor 5
DRP1: Dynamin-related protein 1
Egr1: Early Growth Response 1
ER: Endoplasmic reticulum
ETC: Electron transport chain
GSH: Glutathione
GSH-Px: Glutathione peroxidase
GSSG: Glutathione disulfide
GST: Glutathione-S-transferase
H_2_O_2_: Hydrogen peroxide
HCO^3−^: Hydrogen carbonate ion
HDPE: High-density polyethylene
HepaRG: Immortalized hepatic cells
HepG2: Liver cancer cell line
HIF-1α/HO-1: Hypoxia-Inducible Factor 1-alpha/Heme Oxygenase 1
HNEpCs: Human nasal epithelial cells
HO-1: Heme oxidase 1
HPAEpiC: Human pulmonary artery epithelial cells
Igkv14-126000: Part of immunoglobulin kappa variable
IL1R1: Interleukin-1 receptor type I
IL-1β: Interleukin-1-beta
IL6: Interleukin 6
IPF: Idiopathic pulmonary fibrosis ()
Ki67: Antigen Kiel 67
Lamb3: Laminin Subunit Beta 3
LC3-II: Microtubule-associated protein light chain 3-II
MDA: Malondialdehyde
LIR LC3: LC3-interacting region
MAP: Mitogen-activated protein
MDA: Malondialdehyde
Mfn2: Mitofusin 2
Microplastics: Plastic particles < 5 mm in diameter
NADPH: Nicotinamide adenine dinucleotide phosphate
Nanoplastics: Plastic particles below 1000 nm (1 µm) or in some cases less than 100 nm in diameter
NF-Κb: Nuclear factor kappa-light-chain-enhancer of activated B cells
NH2-PS: Amino-functionalized polystyrene nanoparticles (positively charged)
NO: Nitric oxide
NPs: Nanoplastic particles
NQO1: NADPH quinone oxidoreductase 1
NRF1: Nuclear respiratory factor 1
NRF2: Nuclear respiratory factor 2
O2•─: Superoxide anion
Opa1: Optic atrophy 1
OXPHOS: Oxidative phosphorylation system
PA: Polyamide (nylon)
Parkin: Parkin
PE: Polyethylene
PET: Polyethylene terephthalate
PGC-1α: Peroxisome proliferator-activated receptor gamma coactivator 1-alpha
PINK1: PTEN-induced putative kinase 1
PP: Polypropylene
PS: Polystyrene
PTEN: Phosphatase and tensin homolog
PVC: Polyvinyl chloride
Scel: Sciellin
SOD: Superoxide dismutase
TFAM: Mitochondrial transcription factor A
THP-1: Tohoku hospital pediatrics-1
TiO2: Titanium dioxide
TNF-α: Tumor necrosis factor-alpha
UFP: Ultrafine particles
Upk3b: Uroplakin 3B
UPRmt: Mitochondrial unfolded proteins
UV: Ultraviolet radiation
Δ*Ψ*: Mitochondrial membrane potential
